# Intertrochanteric curved varus osteotomy for subchondral fracture of the femoral head: a case series

**DOI:** 10.1186/s42836-023-00202-6

**Published:** 2023-09-05

**Authors:** Keiji Otaka, Yusuke Osawa, Yasuhiko Takegami, Taisuke Seki, Shiro Imagama

**Affiliations:** 1 Department of Orthopaedic Surgery, Nagoya University Graduate School of Medicine, Nagoya, 466-8550 Japan; 2 Department of Orthopaedic Surgery, Japanese Red Cross Aichi Medical Center Nagoya Daiichi Hospital, Nagoya, 453-8511 Japan

**Keywords:** Intertrochanteric curved varus osteotomy, Subchondral fracture of the femoral head, Young patients, Preoperative intact ratio

## Abstract

Although favorable results have been reported with total hip arthroplasty, joint-preserving treatment should be the first choice for subchondral fracture of the femoral head (SFF) in young patients. This study reviewed four young male patients with SFF who underwent intertrochanteric curved varus osteotomy (CVO). The patients had a mean age of 32.3 years (range: 18–49 years). Conservative treatment was initially attempted in all cases, but failed to alleviate the pain, leading to surgical intervention at an average time of 6 months (range: 4–10 months) after symptom onset. As the fracture sites were located medial to the lateral edge of the acetabulum in all cases, CVO was performed to achieve a postoperative intact ratio of ≥ 34% in the weight-bearing region of the femoral head. The average follow-up period after surgery lasted 4.3 years (range: 2–7 years). Clinical and radiographic assessments were performed pre- and postoperatively. At the latest follow-up, the mean Harris hip score improved from 67.3 preoperatively to 99.5 postoperatively. The average preoperative intact ratio of the weight-bearing region of the femoral head was 12.3%, which increased to 44.3% postoperatively. No progression to femoral head collapse or joint space narrowing was observed on the plain radiographs. CVO is a simple, less-invasive, and beneficial approach for treating SFF in young patients whose fractures occur medial to the lateral edge of the acetabulum.

## Introduction

Subchondral fracture of the femoral head (SFF) is often caused by minor trauma, and when bony fusion is not achieved, the femoral head progressively collapses, the pain worsens, and the quality of life is significantly reduced [1, 2]. Most SFF cases occur as insufficiency fractures in elderly patients with osteoporosis and in post-renal transplant patients [3–5]. However, in young adults, SFF occurs as a fatigue stress fracture due to repeated loading to the hip joint, such as in military training [6, 7]. In addition, SFF has been reported in adults with no history of trauma [1, 8, 9].

**Fig. 1:**
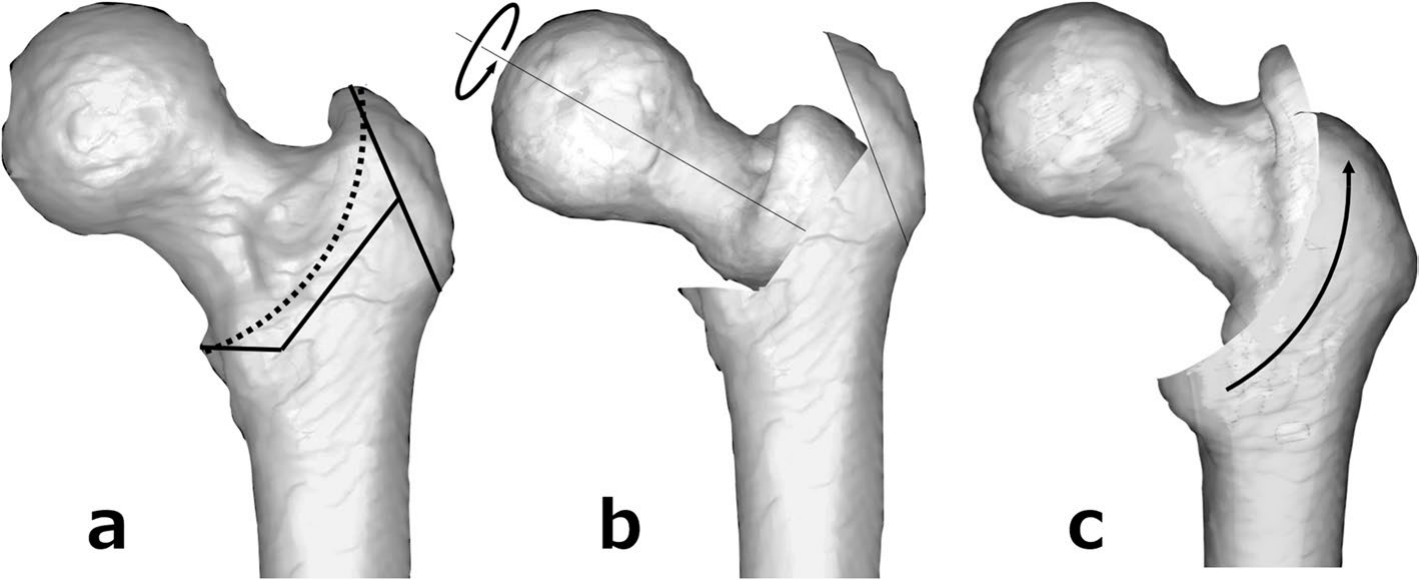
**a** Osteotomy lines; trans-trochanteric anterior rotational osteotomy (ARO) (solid line) and inter-trochanteric curved varus osteotomy (CVO) (dotted line). **b** ARO. The greater trochanter is osteotomized, and the joint capsule is circumferentially incised. The femoral head is rotated anteriorly. **c** CVO. A curved osteotomy is performed from the proximal part of the greater trochanter to the lesser trochanter, and the femoral head is rotated into a varus position

The first treatment for SFF is conservative therapy (avoiding weight-bearing). Conservative treatment is successful in approximately half of the cases, and surgical treatment is considered in cases of persistent pain or progressive femoral head collapse [10, 11]. Total hip arthroplasty (THA) and bipolar hip arthroplasty are indicated in middle-aged to elderly patients. However, THA for young patients is controversial because of the possibility of future revision [12–14]. Therefore, joint-preserving treatment is considered for SFF in young patients whenever possible. Good results have been reported for trans-trochanteric anterior rotational osteotomy (ARO) as a joint-preserving treatment for subchondral insufficiency fracture of the femoral head (SIF) in young adults [15, 16]. However, ARO is a more invasive and complex procedure because it requires trochanteric osteotomy and dissection of the muscle around the hip joint [17, 18] (Fig. 1a, b).

Intertrochanteric curved varus osteotomy (CVO) was originally devised as a treatment for acetabular dysplasia [19]. It has also been adapted for osteonecrosis of the femoral head (ONFH), and many good clinical results have been reported [20, 21]. CVO is technically simple, relatively less invasive in relation to the abductor’s muscles, and less likely to cause leg-length discrepancy [17, 18, 20, 22] (Fig. 1a, c). In this study, we reviewed four cases of SFF in young patients in which good outcomes were achieved with CVO.

## Patients and methods

We retrospectively reviewed four young patients diagnosed with SFF who underwent CVO between April 2010 and December 2021 (Table 1). All patients were men, with a mean (range) age of 32.3 (18–49) years and a mean body mass index of 23.9 (20.4–25.2) kg/m^2^), followed up for 4.3 (2–7) years on average. Two patients developed unexplained hip pain, one developed pain while transporting heavy objects, and one was a high-school student who developed chronic hip pain during baseball practice. None of the patients had a history of steroid administration or excessive alcohol consumption. All cases were diagnosed using magnetic resonance imaging (MRI), and T1-weighted imaging showed a low-signal area in the subchondral bone of the weight-bearing region of the femoral head. The lesions were located medial to the lateral edge of the acetabulum. All patients were first treated non-surgically by precluding weight-bearing and administering non-steroidal anti-inflammatory drugs (NSAIDs). However, the pain persisted in all patients, and two of them showed progression of femoral head collapse. CVO was performed at an average of 6 (4–10) months after onset to improve pain and prevent progressive collapse.

**Table 1:** Clinical findings in the four patients

Patient number	Gender	Age (years)	Intact ratio (%)		Harris hip score		Postoperative LLD (mm)	Follow up (years)
			Preoperative	Postoperative	At first visit	At the latest follow up		
1	Male	30	6	40	64	100	9	2
2	Male	18	0	41	72	100	17	2
3	Male	32	24	51	69	98	11	7
4	Male	49	19	45	64	100	9	6

*LLD* Leg-length discrepancy

The basic surgical plan and technique followed previously reported methods [20]. The surgical plan aimed to obtain a > 34% intact ratio of the postoperative weight-bearing region of the femoral head based on preoperative anteroposterior radiographs of the hip in maximum abduction. A 15 cm longitudinal skin incision was made over the greater trochanter with the patient positioned laterally. After splitting the fascia lata and gluteus maximus, the greater and lesser trochanters were posteriorly exposed by internally rotating the hip joint. The iliopsoas muscle was partially dissected from the lesser trochanter, whereas the external rotators and quadratus muscles were preserved to protect the medial femoral circumflex artery. Curved osteotomy was performed using a reciprocating saw, following the specific crescentic sliding guide, from the greater trochanter to the lesser trochanter. The anterior periosteum was dissected along the osteotomy line, and the femoral neck was repositioned in a varus position by cranial displacement. After confirming that we had obtained an intact ratio of > 34% on fluoroscopy, osteosynthesis was performed using a compression hip screw. Postoperatively, range of motion exercises and 10-kg partial weight-bearing was allowed, and full weight-bearing was allowed after 8–10 weeks.

**Fig. 2:**
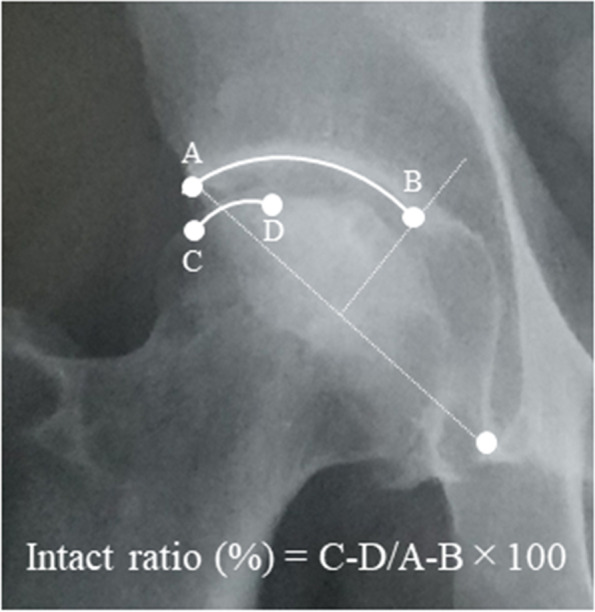
Measurement for the intact ratio to the weight-bearing surface of the acetabulum

We examined the medical records to determine the operative time and intraoperative blood loss, and complications such as infection, peri-implant fracture and nerve palsy. The hip function was assessed using the Harris Hip Score (HHS) preoperatively and at the final follow-up. Radiographic evaluations were performed at 1, 3, and 6 months and every year thereafter. We evaluated the pre and postoperative intact ratio of the postoperative weight-bearing region of the femoral head with anteroposterior radiographs. We also assessed the postoperative progression of the femoral head collapse and arthropathic changes. The intact ratio was calculated by dividing the intact area of the femoral head by the weight-bearing area of the acetabulum on the anterior and posterior radiographs [16] (Fig. 2).

## Results

The mean operative time and intraoperative blood loss were 110.9 ± 29.2 min and 217 ± 101 g, respectively. A mean leg length discrepancy (LLD) of 11.5 mm was observed, but no other complications were found. Hip pain resolved in all patients 1 year postoperatively. Bone fusion was confirmed in all cases within 6 months after surgery, and none of the cases showed postoperative complications. In three of the four cases, T1-weighted MRI at 1 year postoperatively showed loss of low-signal areas of the femoral head. In the remaining case, MRI was not performed postoperatively; however, CT at 1 year postoperatively exhibited fusion and remodeling of the fractured area.

The mean preoperative Harris hip score of 67.3 improved to 99.5 postoperatively. The average preoperative intact ratio was 12.3%, which rose to 44.3% postoperatively. At the current mean follow-up of 4.3 years postoperatively, progression of arthropathic changes and femoral head collapse were not observed on radiographs in any case.

### Report of case 4

**Fig. 3:**
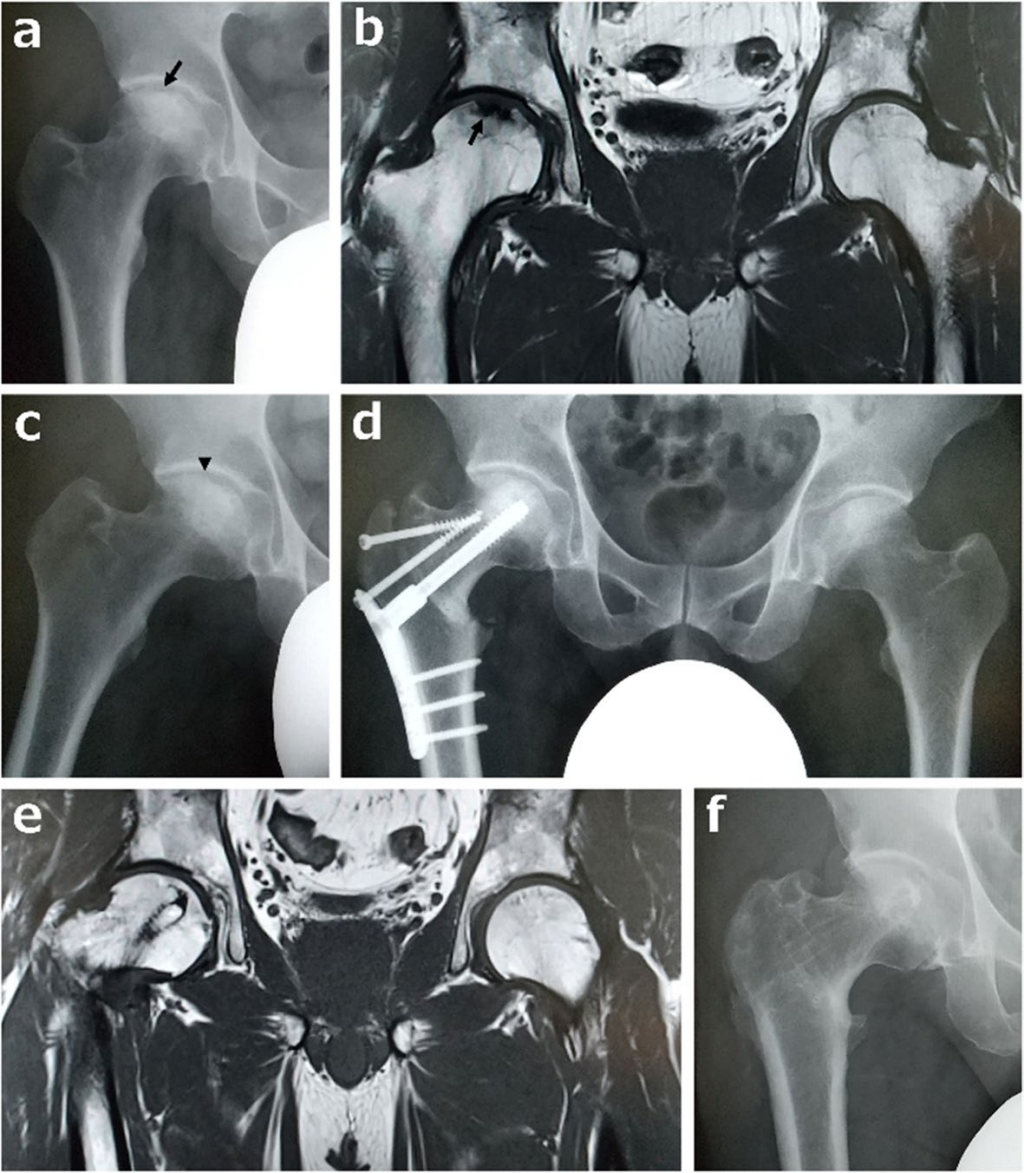
Case 4 (a 49-year-old man). **a** An anteroposterior (AP) radiograph of the right hip at the time of onset shows the collapse of the weight-bearing region of the right femoral head (arrow). **b** T1-weighted magnetic resonance image (MRI) shows a low-signal area and low-intensity band (arrow) in the weight-bearing region of the right femoral head. **c** Preoperative AP radiograph of the right hip in maximum abduction. The arrowhead indicates the lateral end of the fractured area. **d** Six months after onset, inter-trochanteric curved varus osteotomy was performed. **e** T1-weighted MRI 2 years postoperatively. The low-signal area that was present preoperatively disappeared. **f** An AP radiograph taken 6 years postoperatively showed fusion of the osteotomy site and non-progression of the femoral head collapse

A 49-year-old man presented to our hospital for pain in the right hip. The cause of the pain was not apparent. He worked at the airport and routinely carried heavy luggage. Initial examination revealed lameness and a limited range of motion due to pain in the right hip. The Harris hip score was 64 with the right hip. There was no history of illness, and the blood test results were within normal ranges. He had no history of heavy alcohol intake or steroid administration. Plain radiographs showed a collapse of the weight-bearing region of the right hip (Fig. 3a). T1-weighted MRI revealed a linear pattern of low signal intensity parallel to the articular surface in the weight-bearing region of the right femoral head and medial to the lateral edge of the acetabulum (Fig. 3b, c). The patient was diagnosed with SFF based on the imaging findings. Conservative treatment with avoidance of weight bearing and administration of NSAIDs was applied for 4 months. However, the pain did not subside. The patient underwent CVO to alleviate hip joint pain and prevent progressive femoral head collapse (Fig. 3d). The preoperative intact ratio improved from 19 to 45% postoperatively. One year postoperatively, the hip pain resolved, the osteotomy site was fused on plain radiographs, and progression of the femoral head collapse was not observed. T1-weighted MRI showed that the low-signal area at the fracture site had disappeared (Fig. 3e). Six years after the surgery, the Harris Hip Score improved to 100 (Fig. 3f).

## Discussion

In this report, we reviewed four cases in which CVO was performed as a joint-preserving treatment for SFF in young patients. To the best of our knowledge, this is the first report evaluating the results of CVO for SFF. This report demonstrated the CVO was effective as a treatment for young patients with SFF, specifically for those whose fractured areas were medial to the lateral edge of the acetabulum.

SFF shares some features with ONFH but has distinct causes. ONFH occurs due to inadequate blood flow to the lesion, whereas SFF is characterized by a fracture with existing blood flow to the affected area, which allows for healing. Therefore, the early diagnosis of SFF before femoral head collapse is crucial. MRI is necessary when SFF is suspected. Both SFF and ONFH display a T1 low-band image of the femoral head on MRI, but ONFH typically exhibits a concave appearance towards the articular surface, whereas SFF displays a band that is parallel or convex to the femoral head articular surface [23]. However, in cases in which differentiation is challenging, contrast-enhanced MRI is performed.

SFF is managed non-surgically by avoiding weight-bearing if the collapse of the femoral head is mild. Non-surgical treatment is successful in only about half of the cases, and surgical treatment is required if the pain persists or if the femoral head collapse progresses [10]. Although good results have been reported regarding THA for SFF in elderly patients, employing THA in young patients is controversial because of the risk of revision in the future [12–14]. Therefore, joint-preserving surgery should be considered whenever possible. Osteotomy was performed to prevent bearing on the fractured area and ensure healing of the fracture. Yamamoto et al. reported good results at 2 years of follow-up in four young adults with SIF who underwent ARO as a joint-preserving treatment for SFF and there was no evidence of further collapse of the femoral head [15]. ARO was devised for ONFH. However, its use has declined recently because of inconsistent success rates (ranging from 17 to 100%) [24–26]. However, because of the smaller lesion size in SFF than in ONFH, Sonoda et al. compared the results of ARO for SIF and ONFH among young adults and reported favorable results with ARO for SIF [16].

CVO was initially designed for acetabular dysplasia, but in recent years, many favorable results concerning CVO for ONFH have been reported [19–21]. The advantage of CVO lies in that it is a simpler and less-invasive procedure than ARO, as it does not require an incision of the joint capsule, whereas ARO necessitates a full circumferential incision of the joint capsule. Lee et al. made a comparison between CVO and trans-trochanteric rotational osteotomy for ONFH and reported that CVO had a shorter operative time and significantly less postoperative osteophyte formation. Therefore, CVO should be considered the primary joint-preserving surgery for young patients because of its simplicity and reduced invasiveness [17]. CVO is believed to improve the biomechanical condition of the femoral head, as demonstrated by Iwase et al., who reported a significant improvement in the CE angle following inter-trochanteric varus osteotomy [27]. Wang et al. evaluated the impact of CVO on stress reduction for the femoral head in ONFH using finite element analysis and found that an increase in the postoperative intact rate led to a reduction in stress on the femoral head [28]. Although CVO is theoretically unlikely to cause LLD, many cases of LLD have been reported. Ikemura et al. examined 36 patients (involving 42 hips) who underwent CVO and reported an average LLD of 13 mm, with LLD being significantly greater in patients experiencing a limp [22]. In our study, we performed CVO in young patients with SFF, and despite the presence of LLD in all patients, we achieved favorable outcomes without a limp.

**Fig. 4:**
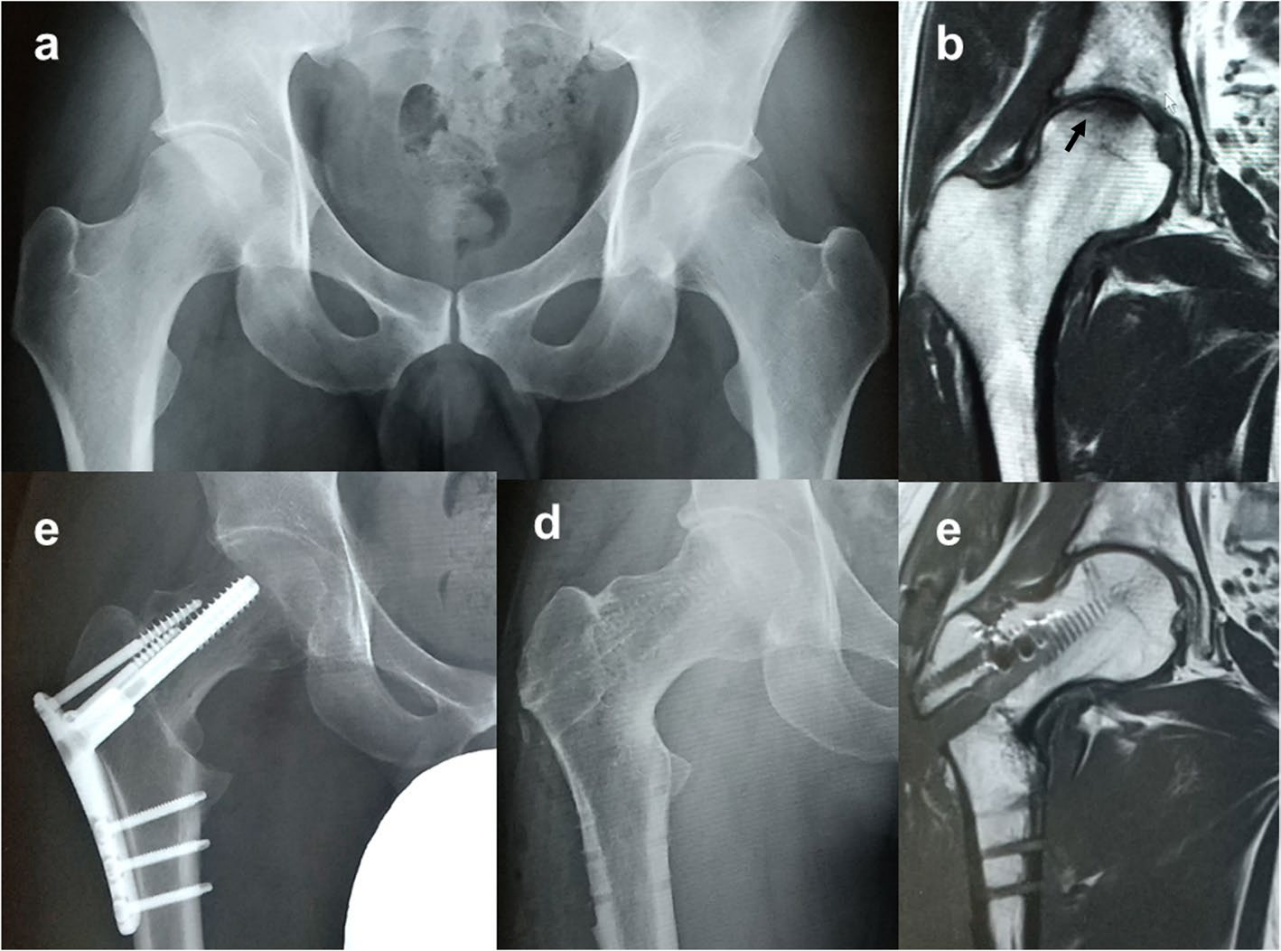
Case 1 (30-year-old man). **a** An AP radiograph of the right hip at the first visit. **b** T1-weighted magnetic resonance image (MRI) shows a low-signal area and low-intensity band (arrow) in the weight-bearing region of the right femoral head. **c** Six months after onset, inter-trochanteric curved varus osteotomy was performed. **d** AP radiograph obtained 2 years postoperatively showing fusion of the osteotomy site. Although there is slight osteophyte formation, there is no narrowing of the joint space, and hip function is good. **e** T1-weighted MRI 2 years postoperatively. The low-signal area that was present preoperatively disappeared

Iwasaki et al. reported that the typical site of SIF is the anterior portion [29]. They proposed two types of SIF. The first is the lateral type caused by contact stress between the acetabular edge and the lateral portion of the femoral head based on insufficient acetabular coverage. The other is the central type in which the fractured area is distributed on the central portion of the femoral head and is independent of the insufficient acetabular coverage. Therefore, ARO, which transposes the posterior intact area to a weight-bearing portion of the joint, may be helpful in many cases of SIF. Conversely, CVO, which rotates the femoral head into a varus position to transport the lateral intact area to a weight-bearing portion, could be indicated in the cases of central type and some cases of lateral type that have healthy articular cartilage on the lateral side of the femoral head. It has been reported that the intact ratio of the postoperative weight-bearing region of the femoral head should be at least 33.3% in patients who undergo CVO for osteonecrosis [30], and it is generally indicated in Types B and C1 of the Japanese Investigation Committee (JIC) classification. Based on this classification, we used CVO for SFF, in which the fractured area was medial to the lateral edge of the acetabulum, and planned to achieve a postoperative intact ratio of ≥ 34% (Fig. 4). ARO should be selected for the cases in which the fractured area is outside the lateral edge of the acetabulum, the range of motion of abduction is poor, or there is difficultly achieving a sufficient intact area.

This study has several limitations. First, we could only recruit a small number of patients, and there was no control group in this study. Second, two patients had a relatively short follow-up period of only two years. Therefore, while many of the fractured areas showed signs of healing, the potential for osteoarthritic progression remained. Finally, the indication for CVO in SFF remains unclear in relation to specific hip joint conditions, such as acetabular dysplasia or femoroacetabular impingement. Okura et al. reported that a CE angle of less than 25° is an independent factor associated with radiographic failure and conversion to THA after CVO for ONFH [30]. A decreased femoral neck-shaft angle has also been implicated in the manifestation of symptoms related to CAM-type FAI [31, 32]. Therefore, CVO may increase the risk of femoroacetabular impingement.

In conclusion, we reviewed four cases of SFF in young patients in whom good outcomes were achieved with CVO. CVO is a simple and less invasive procedure that is useful for SFF in young patients with fractured areas medial to the lateral edge of the acetabulum.

## Data Availability

This study was carried out in the Hospital of the Nagoya University Graduate School of Medicine. The datasets used and/or analyzed during the current study are available from the corresponding author on reasonable request.
